# Determining the effectiveness of fibrin sealants in reducing complications in patients undergoing lateral neck dissection (DEFeND): study protocol for a randomised external pilot trial

**DOI:** 10.1186/s40814-020-00618-w

**Published:** 2020-05-26

**Authors:** Mandeep S. Bajwa, Stacey Carruthers, Rob Hanson, Richard Jackson, Chris Braithwaite, Mike Edwards, Seema Chauhan, Catrin Tudur Smith, Richard J. Shaw, Andrew G. Schache

**Affiliations:** 1 Liverpool Head and Neck Centre, University of Liverpool and Aintree University Hospital NHS Foundation Trust, Liverpool, UK; 2 Liverpool Cancer Trials Unit, University of Liverpool, Liverpool, UK; 3 Department of Biostatistics, University of Liverpool, Liverpool, UK

**Keywords:** Fibrin tissue adhesives, Neck dissection, Postoperative complications, Clinical trial protocol, Pilot studies, Surgical oncology

## Abstract

**Background:**

Complications after major surgery are a significant cause of morbidity and mortality. Neck dissection is one of the most commonly performed major operations in Head and Neck Surgical Oncology. Significant surgical complications occur in approximately 10–20% of all patients, increasing to 40% in patients who have had previous treatment to the area or have multiple co-morbidities and/or polypharmacy.

Current evidence suggests that fibrin sealants (FS) may have potential clinical advantages in Head and Neck Surgery through the reduction of complications, volume of wound drainage and retention time of the drains. However, a paucity of high-quality trial-based evidence means that a surgical trial to determine the effectiveness of FS in reducing the rate and severity of complications in patients undergoing lateral neck dissection is warranted. The DEFeND randomised external pilot trial will address critical questions on how well key components of the proposed study design work together as well as the feasibility of a future phase III trial.

**Methods:**

The study design that is being piloted is that of a two-arm, parallel group, superiority trial with block randomisation in a 1:1 allocation ratio. The interventional arm will constitute the application of FS (Artiss, Baxter Healthcare Ltd.) to the surgical wound following completion of a neck dissection procedure, in addition to standard of care (SOC). The control arm will constitute SOC alone. Eligible patients will include patients who require a lateral neck dissection with a minimum of three cervical nodal levels. Patients who require bilateral neck procedures or undergoing immediate reconstruction with free or regional flaps will be excluded. The outcomes being assessed will be recruitment rate, screened to randomisation rate, fidelity of blinding process using blinding indices, number of missing or incomplete data entries, number of protocol deviations and number of losses to follow-up. Suitability of the outcome measures proposed for the future phase III trial will also be assessed.

**Discussion:**

The anticipated challenges for this study will be recruitment, complexity of the intervention and adherence to the protocol. The outcomes will inform the design, feasibility and conduct of a future phase III surgical trial.

**Trial registration:**

First participant randomised: November 06, 2018; UKCRN Portfolio ID: 37896; ISRCTN99181100.

## Introduction

### Background and rationale

Complications after major surgery are a significant cause of morbidity and mortality and have been shown to have a negative impact on long-term quality of life and psychosocial well-being [1, 2]. In surgical oncology, complications can also delay adjuvant treatment (e.g. radiotherapy) which is known to adversely affect survival [3]. Following resection of the primary tumour, neck dissection is the most commonly performed ‘major operation’ in Head and Neck Surgical Oncology. Hospital Episode Statistics (HES) suggest that approximately 1500 neck dissections are performed within the National Health Service (NHS) in England annually [4]. Significant surgical complications occur in approximately 10–20% of patients undergoing neck dissection [5, 6]. Such risks increase to 40% in patients who have had previous chemo-radiotherapy to the area or when operating on higher risk patients of increasing age, with multiple co-morbidities and/or polypharmacy [7, 8]. Common surgical complications include haematoma formation, surgical site infection, wound breakdown/dehiscence and fistula formation. Management of these complications is frequently painful, invasive and may involve return(s) to theatre. This inevitably delays recovery, which in turn may result in prolonged hospital stay and immobility; both of which are known risk factors for additional complications such as lower respiratory tract infections and venous thromboembolism.

The direct impact on patients of complications following neck dissection has been borne out by a ‘core information set’ for informed consent to surgery for oral or oropharyngeal cancer [9]. Using the Delphi method, this study found details of major or common complications including pain, swelling and bleeding that may require a return to the operating theatre; the likelihood of wound problems; and details of drips, drains and tubes were important to both patients and healthcare professionals alike [9]. Patient opinion is further supported by robust data from a meta-analysis on the use of surgical drains in thyroid surgery demonstrating that they (drains) increased both post-operative pain and infection rates [10]. Clearly drains serve an important role in preventing potentially life-threatening complications due to neck swelling; however, reduction in the duration of their use, through early safe removal, and in reduction of wound-related complications will clearly translate to significant patient benefit in the immediate post-operative period.

A recent systematic review and meta-analysis on the use of fibrin sealants (FS) in Head and Neck Surgery found potential clinical advantages to both patients and healthcare organisations through reduction in complications and volume of wound drainage, thereby minimising the retention time of the drains [11]. FS are commercially available, US Food and Drug Administration (FDA) approved, products that have been investigated broadly across several areas of surgery [12]. FS is applied to the raw surfaces of the surgical wound prior to closure providing an adjunct to haemostasis. The mechanism of action is through replication of the final stages of the clotting cascade through which thrombin cleaves fibrinogen to form a fibrin clot. The subsequent clot effectively seals small vessels and occludes cavity dead space by adhering the wound surfaces; both essential steps in avoiding haematoma formation that may compromise surgical site healing. Results of previous investigations of FS effectiveness in surgery have been variable and have frequently been unduly influenced by poor study design.

The key relevant findings of the systematic review and meta-analysis on the use of FS in Head and Neck Surgery were as follows [11]:

- There is a paucity of high-quality trials on the use of FS in Head and Neck Surgery.
- There was a tendency for FS to reduce drainage volume.
- There was a suggestion that FS may reduce ‘mean retention time of drains’ and ‘hospital length of stay’ but these were not statistically significant.
- Whilst not reaching statistical significance, FS may be protective against complications compared to standard of care. The benefit of FS was greater with regard to haematoma/seroma formation.
- Patients at high risk of complications (e.g. anticoagulation and previous surgery or radiotherapy) were excluded from all studies analysed, leaving the effects of FS in populations most likely to benefit not assessed.
- The role of FS in lateral neck dissection is an area of need for further studies. Only 2 trials have been performed so far that have randomised 78 patients between them [13, 14]. Their inclusion criteria and findings varied greatly and substantial statistical heterogeneity impaired conclusive results in the meta-analysis.

Drawing upon the available evidence and apparent surgical equipoise with respect to FS usage, it is felt that a surgical trial to determine the effectiveness of FS in reducing the rate and severity of complications in patients undergoing lateral neck dissection is warranted. This important clinical question is framed by patient opinion and guided by a clinical desire to reduce morbidity. However, given the difficulties in the delivery of Head and Neck Surgical Trials [15], this external pilot study will be used to answer critical questions on how well key components of the proposed study design work together as well as the feasibility of the future trial.

### Objectives

The key objectives of this randomised external pilot study are to assess the following points:

- Whether patients can be recruited and retained at a rate of approximately 4 patients per month across the 2 centres.
- Determining the effectiveness of the blinding strategy using blinding indices.
- Ensuring the administrative processes of randomisation, allocation concealment and data management work well within the study.
- Assess adherence to the conditions of the protocol.
- Provide evidence to inform the sample size calculation for the future phase III multicentre randomised trial

## Methods/design

### Trial design

This is a randomised external pilot trial set within two UK hospitals offering tertiary head and neck surgery services (Aintree University Hospital NHS Foundation Trust & Queen Victoria Hospital NHS Foundation Trust). Both institutions serve a large population with a mix of both urban and rural communities. Aintree University Hospital treats a population that includes the most deprived 10% of the UK [16]. The target population for this study is patients that are due to undergo lateral neck dissection as part of their treatment for Head & Neck Cancer.

**Fig. 1:**
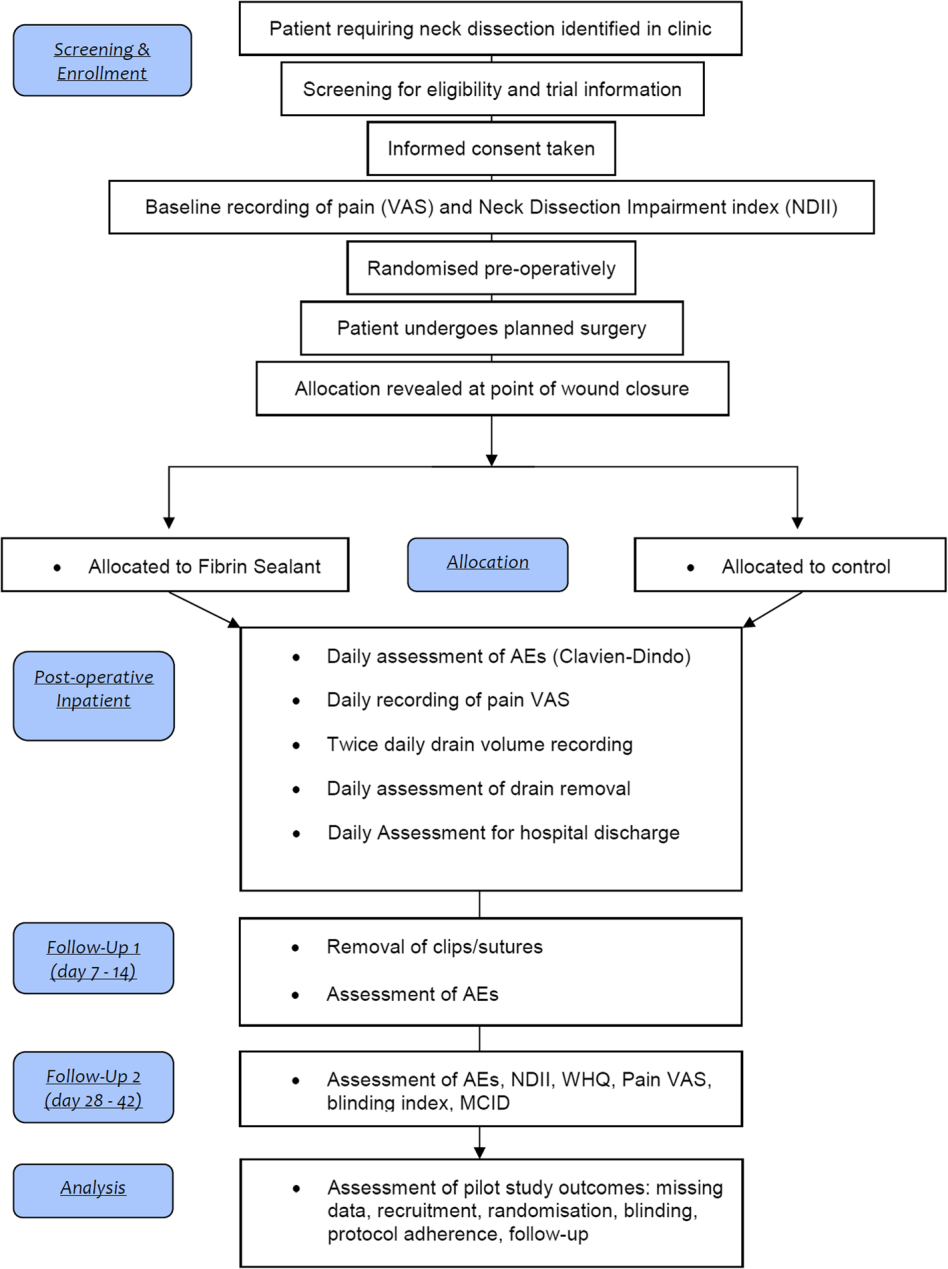
Study flow diagram (CONSORT diagram)

The study design that is being piloted is that of a two-arm, parallel group, superiority trial with block randomisation in a 1:1 allocation ratio. The interventional arm will constitute the application of FS (Artiss, Baxter Healthcare Ltd.) to the surgical wound in addition to standard of care (SOC), and the control arm will constitute SOC alone. For the purposes of this study, SOC constitutes the surgeon performing the neck dissection as they normally would and establishing complete haemostasis. Patients in both arms will have a single surgical drain placed and the wound closed with resorbable sutures across the platysma layer and metal clips to close the skin. The use of FS or any other adjunct to haemostasis in neck dissection is not a common place within the UK. For this reason, the selection of SOC as the comparator is justified. The Consolidated Standards of Reporting Trials (CONSORT) diagram is shown in Fig. 1.

The protocol has been prepared in line with the Standard Protocol Items: Recommendations for Interventional Trials (SPIRIT) guidelines. The SPIRIT Figure shows the different data collection steps of the pilot trial (Table 1); a completed SPIRIT checklist is available as an additional file (Additional file 1).

**Table 1:** Schedule of assessments (SPIRIT figure)

Procedures	Head and Neck Clinic/MDT	Screening	Pre-operative assessment	Baseline*	Day of surgery (day 0)	Follow-up schedule				Premature discontinuation
						Daily in-patient assessment	Follow-up 1 (days 7–14)	Follow-up unscheduled	Follow-up 2 (days 28–42)	
Identify potential participant	X	X								
Approach potential participant to discuss study	X	X								
Medical history		X								
Physical examination		X								
Assessment of eligibility criteria		X								
Review of concomitant anticoagulant medications		X	X	X	X	X	X	X	X	X
Review of previous treatment to the ipsilateral neck		X								
Demographic assessment		X								
Signed consent form				X						
Randomisation				X						
Assessment of patient-reported outcome measures										
Neck pain (VAS)				X		X	X	X	X	X
Neck Dissection Impairment Index (NDII)				X					X	X
Wound Healing Questionnaire (WHQ)									X	X
Surgical protocol										
Neck dissection surgery					X					
Allocation revealed at point of wound closure					X					
Prepare and administer ARTISS (interventional arm only)					X					
Assessment of clinical outcome measures										
Assessment of AEs (Clavien-Dindo)					X	X	X	X	X	X
Wound drainage volume (ml)						X				
Wound drain removal						X				
Hospital discharge						X				
Assessment of pilot study outcomes										
Assessment of blinding strategy									X	X
Assessment of minimal clinically important Difference									X	X
Laboratory tests										
Full blood count**				X						
INR & APTT				X						
Pregnancy test (women of childbearing age)				X						
Histological lymph node yield									X	

*VAS* visual analogue scale, *INR* international normalized ratio, *APTT* activated partial thromboplastin time

*At baseline, all procedures should be done before study intervention

**Full Blood Count must include haemoglobin concentration, platelet count and white cell count

### Trial registration and governance

The DEFeND study was prospectively registered with the ISRCTN registry. ISRCTN99181100 was assigned on 16 May 2018. DEFeND is also registered on the UK Clinical Research Network (UKCRN) study portfolio (Protocol Number: 37896). The University of Liverpool is the sole sponsor for this study (sponsor@liverpool.ac.uk) and has responsibility for trial oversight, indemnity, monitoring trial conduct and governance. All items from the World Health Organization Trial Registration Data Set can be found at 10.1186/ISRCTN99181100.

The funder and sponsor have no role in the study design, data collection, trial management, analysis of results or dissemination. The Research Ethics Committee (REC) agreed that an Independent Data Monitoring and Safety Committee is unnecessary. A Trial Management Group monitors progress approximately monthly. The Trial Steering Committee (TSC) will be convened at the start, mid-point and end of recruitment.

### Eligibility criteria

Inclusion criteria:

• Patients due to undergo lateral neck dissection

• Neck dissection to include a minimum of 3 levels [17]

• Patients who have the capacity to consent

Exclusion criteria:

• Age < 18 years

• Bilateral neck dissection

• Presence of a vascular pedicle for reconstruction

• Pregnancy or breastfeeding

• Known hypersensitivity reaction to aprotinin

• Previous exposure to FS within 6 months

• Known allergy to dairy products

### Storage, preparation and administration of fibrin sealant (FS)

The DEFeND study will use the 2-ml pre-filled double-chamber syringe preparation of “Artiss” FS manufactured by Baxter Healthcare LTD. Artiss has a shelf life of 2 years and should be stored in its protective packaging and transported in a frozen state at less than – 20 ^o^C [18].

The “Quick thawing” technique, as described by the manufacturer, will be used to prepare the FS for use. Quick thawing is done by placing the FS in a sterile water bath at 33 °C to a maximum of 37 °C for 5 min. An infrared thermometer is used to check the water temperature prior to immersing the FS. Once thawed, the FS may be stored at 33–37 °C for a maximum of 4 h. Inspection of both chambers after thawing should reveal clear or slightly opalescent viscous liquids. Solutions that are cloudy/discoloured, contain deposits/particulate matter or solidified should be discarded [18].

The FS is delivered into the wound as a fine spray driven by medical grade air. The “EasySpray” pressure regulator device is setup as per the manufacturer’s instructions (Baxter Healthcare LTD) and the spray pressure set to 1.5 bar. The scrub practitioner uses the Sprayset tubing to attach the FS syringe to the EasySpray pressure regulator. Precise details on how this is done can be found on the manufacturer’s website (http://www.baxterspraysafety.com/uk.html).

The administration of FS requires at least 3 people including a scrub practitioner, assistant and surgeon. While the FS is being thawed, the surgeon should irrigate the wound with 100 ml of sterile normal saline, dry the wound with sterile gauze swabs, secure the surgical drain and place several resorbable parachute sutures (4–6) across the platysma layer. These sutures should be loosely clipped and not tied to ensure good access to the wound. The drain should be held temporarily outside of the wound to ensure the perforations are not occluded by the FS. The prepared Sprayset should not be held any closer than 10 cm to the wound to avoid the risk of air embolism. Once the administration of FS has commenced, the surgeon has 60 s to deliver up to 2 ml and manipulate the skin flaps into position prior to polymerisation. It is therefore important to strictly adhere to the time using a stopwatch. The assistant should retract any structures (e.g. sternocleidomastoid muscle) to ensure the surgeon can reach sheltered areas and administer the FS evenly in a thin layer across the entirety of the wound. Once the administration is complete, the drain and skin flaps are repositioned and even pressure applied to the wound (using a large rolled-up gauze swab) while the surgeon ties off all of the parachute sutures. It is very important that the surgeon does not lift the skin edges up while tying the sutures as this may break any adhesive bond between tissue layers. The surgical vacuum drain should then be activated, and the assistant should maintain pressure on the neck for a full 3 min. After 3 min, clips/staples are used to close the skin edges. When spraying the FS, changes in blood pressure, pulse, oxygen saturation and end-tidal CO_2_ should be monitored because of the possibility of air embolism.

### Outcomes

The proposed outcome measures for this study can be divided into those that are specific to the pilot study and those informing any future phase III trial seeking to determine the effectiveness of FS in neck dissection. As this is a pilot study, formal assessment of efficacy, cost or safety across treatment arms will not be made.

The outcomes for the pilot study include the following:

- Proportion of eligible patients recruited to the study, calculated as the screened to randomisation rate.
- Reasons for failure to screen potentially eligible patients.
- Recruitment rate measured as the number of patients randomised each month.
- Reasons for failure to randomise.
- Reasons for failure to reveal allocation at a specific time point during surgery.
- Fidelity of the blinding process (both patients and outcome assessors) as detected by blinding indices.
- Accuracy of data recording, summarised by the number of key data items with missing/incomplete data entries.
- Number of patients lost to follow-up.
- Protocol adherence, measured by the number of major/minor protocol deviations observed through the study.
- Determining the minimal clinically important difference (MCID) in clinical endpoints by questioning recruited patients and recruiting clinicians.

The clinical outcomes to inform a future phase III trial include the following:

- Clavien-Dindo classification of surgical complications [19].
- Twice daily wound drainage volume (ml).
- Time (hours) for daily wound drainage volume to reach < 1.25 ml/h.
- Time (hours) to drain removal (as dictated by drainage volume).
- Total wound drainage volume (ml).
- Time (hours) to be declared medically fit for hospital discharge and time (hours) to actual hospital discharge.
- Incremental cost-effectiveness ratio.

Patients reported outcomes to inform the future phase III trial include the following:

- Neck Dissection Impairment Index (NDII). This is a procedure-specific patient-reported outcome measure [20].
- Daily patient-reported pain score using visual analogue scale (VAS).
- Wound Healing Questionnaire (WHQ). This is a questionnaire currently in the process of validation to assess wound healing after surgery [21].

### Clavien-Dindo classification of surgical complications

The Clavien-Dindo classification is a widely accepted tool to grade the severity of surgical complications (Table 2) [19]. Because it is a generic classification, grading can be open to interpretation when applying it to complications specific to Head and Neck Surgery. For example, Monteiro et al. found that there was imperfect inter-observer reliability in scenarios where patients underwent a surgical procedure that did not require returning to the operating theatre [22]. To avoid this issue within the context of the DEFeND study, the severity of common/established complications associated with neck dissection has been graded to conform to the Clavien-Dindo classification (Table 3). An assessment of complications using the Clavien-Dindo classification will be carried out at every patient encounter after surgery, i.e. every day of the patient’s hospital stay and at subsequent scheduled and unscheduled follow-up visits.

**Table 2:** Clavien-Dindo classification of surgical complications [19]

Grade of complication	Definition
**Grade I**	Any deviation from the normal postoperative course without the need for pharmacological treatment or surgical, endoscopic and radiological intervention. Acceptable therapeutic regimens are drugs such as antiemetics, analgesia, diuretics and electrolytes and physiotherapy. This grade also includes wound infections opened at the bedside.
**Grade II**	Requiring pharmacological treatment with drugs other than those allowed for grade I complications. Blood transfusions and total parenteral nutrition are also included.
**Grade III**	Requiring surgical, endoscopic or radiological intervention.
Grade III-a	Intervention NOT under general anaesthesia
Grade III-b	Intervention under general anaesthesia
**Grade IV**	Life-threatening complication requiring HDU/ICU management
Grade IV-a	Single organ dysfunction (including dialysis)
Grade IV-b	Multi-organ dysfunction
**Grade V**	Death

*HDU* high dependency unit, *ICU* intensive care unit

**Table 3:** Common head and neck/general complications conformed to the Clavien-Dindo classification

Post-operative complication	Description of severity	Clavien-Dindo grade
Neck wound infection	Localised and superficial to platysma, e.g. stitch abscess	**I**
	Spreading cellulitis or superficial wound infection with no underlying collection treated with antibiotics	**II**
	Collection deep to platysma requiring drainage (not under GA)	**IIIa**
	Collection deep to platysma requiring drainage (under GA)	**IIIb**
	Large collection with organ and/or life-threatening sequelae (i.e. airway obstruction, severe sepsis, septic shock)	**IV** (**a** or **b** depending on organ dysfunction)
Other surgical site infection	Localised infection requiring topical or non-invasive treatment	**I**
	Infection requiring treatment with antibiotics only	**II**
	Collection requiring drainage (not under GA)	**IIIa**
	Collection requiring drainage (under GA)	**IIIb**
	Large collection with organ and/or life-threatening sequelae (i.e. airway obstruction, severe sepsis, septic shock)	**IV** (**a** or **b** depending on organ dysfunction
Bleeding/haematoma	Haematoma not requiring drainage or suitable for simple aspiration with a needle (not radiologically guided)	**I**
	Need for blood transfusion	**II**
	Requiring drainage (not under GA). Includes radiologically guided aspiration/drainage	**IIIa**
	Requiring drainage or return to theatre for haemostasis (under GA)	**IIIb**
	Haematoma/haemorrhage sufficiently large to obstruct airway or cause hypovolaemic shock	**IV** (**a** or **b** depending on organ dysfunction)
Chyle leak	Low output leak (< 500 ml/24 h) suitable for low-fat diet and compression only	**I**
	Requirement for pharmacological management including total parenteral nutrition	**II**
	Radiologically guided occlusion	**IIIa**
	Return to theatre for the procedure under GA	**IIIb**
	Evidence of end-organ dysfunction	**IV** (**a** or **b** depending on organ dysfunction)
Wound breakdown	Superficial skin dehiscence (platysma layer intact) managed with dressings	**I**
	Small fistula managed by an enteral tube or parenteral nutrition only	**II**
	Deep dehiscence (through platysma layer) or fistula managed with procedure not under GA	**IIIa**
	Deep dehiscence (through platysma layer) or fistula managed with the procedure under GA	**IIIb**
	Evidence of end-organ dysfunction	**IV** (**a** or **b** depending on organ dysfunction)
Seroma/sialocele	Small collection not requiring drainage or suitable for aspiration with a needle (not radiologically guided)	**I**
	Salivary fistula managed medically (e.g. anticholinergic)	**II**
	Requiring drainage (not under GA). Includes radiologically guided aspiration/drainage	**IIIa**
	Requiring re-exploration and/or drainage (under GA)	**IIIb**
	Large collection obstructing airway	**IVa**
Hypersensitivity	Mild reaction not requiring treatment	**I**
	Mild/moderate/severe reaction treated with medication (e.g. antihistamine and/or steroid and/or adrenaline)	**II**
	Anaphylactic shock	**IV** (**a** or **b** depending on organ dysfunction)
Air embolism	By definition clinically evident air embolism results in cardiorespiratory dysfunction	**IVb**
Pneumothorax/haemothorax	Small pneumothorax managed without a chest drain	**I**
	Pneumothorax/Haemothorax without respiratory failure requiring chest drain	**IIIa**
	Evidence of respiratory failure or any other organ dysfunction	**IV** (**a** or **b** depending on organ dysfunction)
Pulmonary embolism	Small PE without evidence of respiratory failure managed with anticoagulation only	**II**
	Evidence of respiratory failure or any other organ dysfunction	**IV** (**a** or **b** depending on organ dysfunction)
Deep vein thrombosis	Managed with anticoagulation only	**II**
	Need for endovascular intervention including filters not under GA	**IIIa**
	Need for endovascular intervention or surgical thrombectomy under GA	**IIIb**
Lower respiratory tract infection (including aspiration)	Managed with physiotherapy only	**I**
	Managed with antibiotics	**II**
	Evidence of respiratory failure or any other organ dysfunction	**IV** (**a** or **b** depending on organ dysfunction)

*GA* general anaesthesia

### NDII

The NDII is a procedure-specific health-related quality of life (HRQoL) assessment tool. The tool is validated for use in patients who have undergone selective or modified radical neck dissection [20]. Patients will undertake a baseline NDII pre-operatively and at follow-up visit 2 (4–6 weeks). Although the NDII is not validated for use 4–6 weeks after surgery, there is evidence that the NDII score at this early juncture is representative of longer-term HRQoL [23].

### WHQ

The WHQ has been developed and validated for use in the Bluebelle study; a feasibility study of different wound dressing strategies for the prevention of surgical site infection in elective and unplanned abdominal surgery [21]. Currently, the WHQ has not been validated for use in Head and Neck Surgery. Patients recruited to the DEFeND study will complete the WHQ at follow-up visit 2 (4–6 weeks). Blinded clinicians will assess the wound using the Centres for Disease Control and Prevention (CDC) criteria of surgical site infection. It is expected that this data will contribute to the validation process.

### Health economics

The health economic (HE) assessment of using FS will be piloted using the ‘incremental cost-effectiveness ratio’ (ICER). This will calculate the average incremental cost associated with each surgical complication prevented when compared to SOC. This will be calculated using the following equation: ICER = ((overall cost of FS arm—overall cost of SOC arm)/(no. of complications in FS arm—no. of complications in SOC arm)).

The variation in costs between the treatment arms will be calculated individually for each patient based on their time in the operating theatre (including returns to theatre), their length of stay within each ward type, their number of hospital visits in the immediate post-operative period (both planned and unplanned), and the cost of materials (including those required to administer FS).

### Sample size

As this is a pilot study, no formal power/sample size calculation based on clinical data is given. For this study, the two main outcomes of interest are to determine accurate estimates of the rate of recruitment (being the number of patients recruited relative to the number eligible) and to collect sufficient clinical data to accurately estimate a sample size for a future study. It is estimated that over the study period, approximately 50 patients will be recruited at rate of 30%. Based on this, 50 patients (25 in each arm) will produce a standard error of approximately 6.5% and a 95% confidence interval of approximately (17–43%) will be obtained. With respect to surgical complication rate, being the clinical outcome of current greatest interest, even if a response rate of 50% is observed, then a 95% confidence interval of (0.36, 0.64) will be observed which provides sufficient precision for a future sample size.

### Recruitment

Patients eligible for the DEFeND trial will be screened through outpatient clinics and weekly Head and Neck Multidisciplinary Team meetings. The steps that will be completed on all patients to ensure they meet enrolment criteria include the following:

1. Clinical examination
2. Detailed medical history including previous treatment/surgery to the head and neck
3. Clinical decision to offer a lateral neck dissection

Screening will be performed upon a patient’s possible eligibility for the study as above and will be documented in a secure online ‘Screening log”’ managed by the Liverpool Cancer Trials Unit (LCTU). There are no restrictions regarding concomitant care or interventions during the trial.

Eligible patients should be approached within the outpatient setting and provided with a full explanation of the trial. The patient should also receive an up to date version of the Patient Information Sheet (PIS) (see Additional file 2). Once the patient has had the opportunity to read the PIS and ask any questions, they may consent to the trial (see Additional file 3). The removal of a (24 h) cool-off period prior to signing the consent form has been agreed by the REC; the patient is simply given as much time as they deem necessary. The rationale for such an approach was to reduce patient burden; the recruiting hospitals are tertiary centres with many patients travel long distances to attend appointments. Harmonising the research process with standard clinical care appointments seeks to avoid extra hospital visits, allowing patients the option to provide study consent on the day of being approached.

The DEFeND trial requires that completed consent forms are securely uploaded to the LCTU’s online portal with the patient’s identifiable data still visible. The consent forms will be checked by two independent members of the central trial team. Once these checks have taken place and the consent considered valid, the uploaded document will be permanently deleted from the portal. Any further reference to the consent will need to be performed by accessing the original hard copy stored at the site in the patient’s medical records.

### Allocation

Randomisation lists will be computer-generated by the statistician prior to the recruitment of the first patient. Patients will be randomised using a 1:1 ratio. Lists will be produced based on the principle of randomly permuted blocks with random block sizes of 2 and 4. Patients will only be stratified according to the hospital in which they receive their treatment.

The process of randomisation will be undertaken pre-operatively using the “Treatment Allocation RanDomIsation System” (TARDIS) software version 3.8. This software has been developed by the LCTU. The allocation will be concealed to everyone including the person performing the randomisation. Once randomisation has been performed, the surgeon caring for the patient will receive an automated email which contains a link to reveal the allocation. Once the patient has undergone their neck dissection immediately prior to the point of wound closure, the surgeon will open the link and login to TARDIS to reveal the allocation. The exact time and date this occurs will be recorded and cross-referenced against the start and finish times of surgery as a quality assurance step to minimise performance bias. The randomisation process can be performed either centrally or at the site once the eligibility criteria have been entered and signed off by the Principal Investigator.

As part of the blinding strategy, any clinicians who will be assessing study outcomes must leave theatre prior to the revealing of treatment allocation. They must not return until the theatre has been cleared of any evidence of FS usage. The surgeon administering the FS will not be allowed to assess study outcomes for the patient and must delegate this responsibility to a suitable colleague.

### Blinding

Patients, clinical outcome assessors, ward staff and research staff both centrally and at the site will be blinded to the allocation. Only members of the surgical team present in the theatre when the allocation is revealed will know which treatment arm the patient has been allocated to. These individuals are not permitted to inform colleagues or assess trial outcomes. The operation note, medical case notes and any other documentation that leaves the operating theatre will not state the allocation. The effectiveness of this blinding strategy will be assessed using blinding indices.

The patient will be unblinded if they suffer a serious adverse event and knowledge of the allocation is required for the ongoing medical management of the condition. It is unlikely that this trial will require unblinding as the FS is administered only once in the theatre environment. A severe hypersensitivity reaction, air embolism or transmission of an infective agent constitute a serious adverse event. If they occur, severe hypersensitivity and air embolism would be anticipated to occur during or immediately after administration in the theatre setting. Staff caring for the patient at this time will not be blinded, so there will not be a delay in diagnosis and emergency management.

In the event that the patient is diagnosed with an infectious disease that was not diagnosed pre-operatively, they will be unblinded. Based on the ‘Serious Hazards of Transfusion’ 2017 annual report [24], the following infectious diseases are known to have been transmitted via blood products in the UK:

1. Hepatitis A, B, C or E
2. Human immunodeficiency virus (HIV)
3. Parvovirus (B19)
4. Cytomegalovirus (CMV)
5. Human T cell lymphotropic virus (HTLV) types I and II
6. Malaria
7. Variant Creutzfeldt-Jakob Disease (vCJD) or any other prion disease

If the patient is newly diagnosed with any of the above infectious diseases, they will be unblinded and immediately referred to the appropriate medical specialists for treatment.

### Data management

Data is entered directly into electronic case report forms (eCRF) by research teams at the site using MACRO v4 database software. Delegated staff who have undertaken training to use the software and submitted their current curriculum vitae (signed and dated) and GCP certificate (dated in the last 3 years) will be given access to log in.

Site staff are expected to input data directly into the eCRF within the clinical environment and in real time. MACRO v4 has been programmed to calculate the rate of wound drainage (millilitres per hour). If the rate falls below 1.25 ml/h, MACRO v4 will recommend removal of the drain in keeping with the protocol. This process will only work if data is entered in real time.

Electronic versions of the patient-reported questionnaires will be stored on the LCTU’s portal. Research staff at the site will have individual logins to access this documentation. It is expected that sites will download the questionnaires and hand them to patients for completion at specific time points as stipulated in the protocol. It is also expected that the responses will be transcribed to the eCRF on the same day as the patient completes them.

To ensure the database is only updated or amended by authorised individuals, the data entry system requires users to have a username and password, to be entered at the start of each data entry session.

Users should log out of the data entry system each time they leave their work station unattended. Each computer station should be set to revert to a password-protected screen saver mode when left idle for 10 min.

Individuals should only work under their own username and should not log anyone else onto the system. The MACRO database package has several methods built in to maximise security:

- Each password must contain between 6 and 15 characters, including lower and upper case letters and at least 1 number.
- If left idle for 20 min MACRO automatically locks out the user, who must then re-enter their password to regain access to the database.

MACRO records any changes made to data in the system, and all user activity is logged. For a particular question, the audit trails consist of a chronological record of the status, response value, warning messages and overrule reasons, reasons for change, comments and lock status.

Whenever any of these items are changed, a record is kept of the date and time, and the username of whoever made the change. Users with appropriate permission have access to view audit trails; however, no-one is able to change or delete an audit trail. The audit trail allows the LCTU to monitor the quality of individual team members’ data input and highlight training needs if consistent errors are found.

### Statistical methods

The primary analysis will be carried out on the full data set and will be based on the intention to treat principle, retaining patients in their initially randomised groups irrespective of any protocol violations.

Missing data are expected to be limited, and final analyses are planned to be carried out on a complete case basis. If substantial missing data (> 10%) are observed in either a study outcome or key prognostic covariate, then multiple imputation using chained equations will be applied.

There are no formal comparisons of treatment groups, and therefore, no levels of significance against which hypotheses will be tested. As a guide however, all results will be reported using nominal 95% confidence intervals.

As this is an external pilot study, all data analyses shall take the form of descriptive statistics. Continuous data shall be summarised as medians with associated inter-quartile ranges and categorical data shall be summarised as frequencies of counts and associated percentages.

In terms of clinical outcomes, aside from descriptive statistics, informal comparisons between allocated groups will be made using the difference in means for continuous covariates and differences in rates for categorical covariates.

### Data monitoring

Formal interim analyses of the accumulating data will be performed at 6 monthly intervals after the recruitment of the first patient. A formal Independent Data Monitoring and Safety Committee (IDMSC) will not be convened. In keeping with the guidance outlined in the document ‘Guideline in Data Monitoring Committees’ published by the Committee for Medicinal Products for Human Use [25], it is thought that an IDMSC is not required. This is because patients will be treated for a very short period of time (single administration during surgery) and fibrin sealants are well characterised and already widely used within healthcare. Although there are potential risks to patients, these are incredibly rare and known.

The independent members of the TSC (chairperson, expert, statistician) will take responsibility for reviewing all interim safety data. The independent members will be asked to give advice on whether the accumulated data from the trial, together with results from other relevant trials, justifies continuing recruitment of further patients or further follow-up. Given this is a pilot/feasibility study, it is anticipated that the TSC will only recommend termination on grounds of safety.

### Safety

Surgical complications and adverse reactions to fibrin sealant that are Clavien-Dindo grade IV or above (see Tables 2 and 3) will be the only events reported to assess safety. As the ‘Clavien-Dindo Classification of Surgical Complications’ constitutes a primary outcome measure for the DEFeND pilot trial, the presence of post-operative complications along with their grade will be recorded on the eCRF. All complications related to the neck dissection surgery and/or use of fibrin sealant that are Clavien-Dindo grade III-b or below that meet the definition of serious are exempt from Serious Adverse Event (SAE) reporting. Such events are ‘expected’ and should only be recorded in the relevant section of the eCRF.

Post-operative complications related to either neck dissection or use of fibrin sealant that are Clavien-Dindo grade IV or above are ‘unexpected’ for the DEFeND trial. The LCTU will notify the main REC of all Suspected Unexpected Serious Adverse Reactions (SUSARs) occurring during the study according to the following timelines; fatal and life-threatening within 7 days of notification and non-life-threatening within 15 days. All investigators will be informed of all SUSARs occurring throughout the study.

Site staff (with the exception of the surgical team) will be blinded; therefore, the SAE reporting form will not state which arm the patient has been randomised to. For the purposes of SAE reporting, it should be assumed that the patient was randomised to receive fibrin sealant (i.e. interventional arm). Causality should be assigned to the following:

1. Anaesthetic
2. Generality of surgery (including surgical airway, primary tumour resection)
3. Neck dissection surgery
4. Use of fibrin sealant

Pregnancy is listed as an exclusion criterion for entry to the DEFeND trial. In the event of a patient becoming pregnant after recruitment to the trial, this fact should be reported in the same way as an SAE. The guiding principles in this event are as follows:

1. If the patient has not yet received treatment, or completed treatment, the patient may be withdrawn from the trial.
2. Once treatment is complete, i.e. the patient is in follow-up phase, it may well be possible to retain the patient to the conclusion of the trial.
3. A decision will be made in the best interests of the patient between the treating clinician and the Chief Investigator as to retention in the trial and any continuing cancer therapy.

### Dissemination policy

The results of the pilot trial will be reported in peer-reviewed journals, in a report to the funder and as a lay summary to participants. We will apply authorship criteria established by the International Committee of Medical Journal Editors.

## Discussion

The key challenges that are anticipated in the DEFeND external pilot trial are common to many surgical RCTs and can be encapsulated within three broad headings: barriers to recruitment, complexity of the intervention and adherence to the protocol.

### Barriers to recruitment

Kaur et al. described the barriers to recruitment for surgical trials in Head and Neck Oncology by conducting a survey of investigators from three of the earliest trials within the National Institute of Health Research (NIHR) portfolio [15]. Given that UK-based surgical trials in Head and Neck were in their relative infancy, all of these trials faced challenges with recruitment. The most commonly perceived barriers were lack of equipoise amongst patients demonstrated by a refusal to participate due to an expressed treatment preference, patient consent refusal owing to aversion to randomisation, excess complexity and amount of information provided to patients and lack of time in the clinic. All of these barriers are related to how investigators communicate the premise of the trial to patients. The ‘Merseyside Head and Neck Cancer Patient Research Forum’ is comprised of patients who have undergone treatment for head and neck cancer and was setup specifically to facilitate patient and public involvement in research. The PIS and informed consent forms (see Additional files 2 and 3) have been successfully passed through the forum to ensure they are easily understood while delivering sufficient detail and conveying equipoise.

Given the increase in complexity of the UK clinical trials, trials with overlapping inclusion criteria might also present a barrier to recruitment. Aintree University Hospital has a very research active Head and Neck Surgery Department, and it is possible that patients will be offered more than one study to participate in. This may result in patients suffering from excessive research burden resulting in consent refusal across the board. A predetermined hierarchy of recruitment has been decided amongst all the CI/PIs of the competing studies to enable investigators to minimise the risk of research burden.

The pilot and feasibility outcomes in the DEFeND external pilot trial will allow an in-depth analysis of recruitment and identify any further barriers. This will inform the design and execution of the phase III trial and attempts to mitigate these barriers will be made.

### Complexity of the intervention

One of the differences between surgical trials and pharmacological trials is the complexity of the intervention. Surgical interventions are multi-faceted and depend on the performance of the wider healthcare team in addition to the skill, experience and decision making of the operating surgeon. Outcomes in the DEFeND study are dependent on the perioperative management of patients; the neck dissection surgery itself; the need for other synchronous surgical procedures e.g. resection of the primary tumour; and the intervention itself. As the administration of FS is considered SOC, it is expected that randomisation will balance the variables equally across both arms.

In order that surgeons administer FS in a standardised way, a video has been produced and laminated sheets hung up in operating theatres providing temporal detail of the protocol. Many surgeons will be experienced at administering FS, but for those that are not ‘ad hoc’, training will be available. It is recognised that surgeon level performance will vary and as such the ideal standard procedure will not be replicated on each occasion. Currently, there are no requirements for surgeons to perform the surgery/administer FS a minimum number of times or achieve a predetermined standard. This is primarily to reflect the ‘real-world’ nature of the study. If the impact of the learning curve is clearly demonstrated in the pilot data, then surgeons acquiring a certain level of expertise to participate in the phase III trial will be considered.

### Adherence to the protocol

While every effort has been made to harmonise the protocol with standard clinical/research practice, there will inevitably be some challenges with adherence.

i) The use of eCRF is new to both research sites and requires real-time data entry. This will require training and logistical planning by sites to ensure there are sufficient research staff available (including weekend cover).
ii) The allocation needs to be revealed at a specific time point during surgery. This requires surgeons to access their email, login to the randomisation software (TARDIS) and reveal the allocation. To minimise performance bias, the allocation must not be revealed too early; this will be monitored by recording the date and time the allocation is revealed and cross-referencing this with the start and finish times of surgery.
iii) The blinding strategy may be difficult to enforce within busy clinical practice. The surgeons present in theatre at the time of the allocation reveal are not allowed to assess trial outcomes. In standard clinical practice, it is routine for the operating surgeon to review their patient post-operatively; eliminating their influence on outcomes including the patient’s perception of outcomes will be difficult. If it is known that a particular surgeon will be reviewing the patient post-operatively, they will be asked to vacate theatre at the time of the reveal.
iv) Patients are required to attend two planned follow-up appointments after surgery. The first at 7–14 days to coincide with the removal of skin clips and the second at 4–6 weeks to coincide with the first routine clinic appointment. It is possible that this schedule will clash with some follow-up arrangements and necessitate patients making extra visits. A modest financial refund for travelling expenses will be offered to patients for any extra visits.

## Progression to phase III trial

This initial study aims to inform the design, feasibility and conduct of a future phase III surgical trial. The decision to progress to this future trial will be based on variables such as recruitment rate, safety of intervention, sample size estimation and ongoing surgical equipoise. It is anticipated that further amendments to the protocol will be required to fine tune the trial design based on the outcomes of this external pilot trial and feedback from sites (both research staff and surgeons). All these issues will be discussed by the TSC once the external pilot trial has closed and a decision to progress to a phase III trial made.

## Trial status

Recruitment started on November 06, 2018, and is on target to achieve at the pre-specified sample size in the scheduled 12 months. The interventions appear to be acceptable to potential participants, and approximately 50% of those who have been approached to take part have given written consent. The protocol being used is version 2.0 dated June 27, 2018.

## Supplementary information

**Additional file 1.** SPIRIT 2013 Checklist: Recommended items to address in a clinical trial protocol and related documents*.

**Additional file 2.** Patient information sheet.

**Additional file 3.** Patient consent form.

## Data Availability

Not applicable.

## Abbreviations

AE: Adverse event
APTT: Activated partial thromboplastin time
CDC: Centre for Disease Control and Prevention
CONSORT: Consolidated Standards of Reporting Trials
eCRF: Electronic case report form
FDA: United States Food and Drug Administration
FS: Fibrin sealant(s)
GCP: Good Clinical Practice
HDU: High-dependency unit
HE: Health economics
HES: Hospital Episode Statistics
HRQoL: Health-related quality of life
ICER: Incremental cost-effectiveness ratio
ICU: Intensive care unit
IDMSC: Independent Data Monitoring and Safety Committee
INR: International normalized ratio
ISRCTN: International Standard Registered Clinical/Social Study Number
LCTU: Liverpool Cancer Trials Unit
MACRO: Electronic data capture software
MCID: Minimal clinically important difference
NDII: Neck Dissection Impairment Index
NHS: National Health Service
NIHR: National Institute of Health Research
PIS: Patient Information Sheets
REC: Research Ethics Committee
SAE: Serious adverse event
SOC: Standard of care
SPIRIT: Standard Protocol Items: Recommendations for Interventional Trials
SUSAR: Suspected Unexpected Serious Adverse Reaction
TARDIS: Treatment Allocation Randomisation Software
TSC: Trial Steering Committee
UKCRN: United Kingdom Clinical Research Network
VAS: Visual analogue scale
WHQ: Wound Healing Questionnaire
