# Supplementary material for: Determining the effectiveness of fibrin sealants in reducing complications in patients undergoing lateral neck dissection (DEFeND): study protocol for a randomised external pilot trial
Source: Pilot Feasibility Stud. 2020 May 26;6:76. doi: 10.1186/s40814-020-00618-w (PMC7251660; doi:10.1186/s40814-020-00618-w)
Supplement: Supplementary file 3 — Additional file 3. Patient consent form. [file 40814_2020_618_MOESM3_ESM.doc]

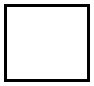

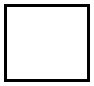


| PATIENT CONSENT FORM (please read carefully) | | | | | |
| --- | --- | --- | --- | --- | --- |
| **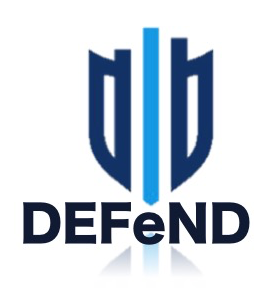**  **Determining the Effectiveness of Fibrin Sealants in Reducing Complications in Patients Undergoing Lateral Neck Dissection:**  **A randomised external pilot trial** | | | | | |
| Name of Researcher:______________________________________ | | | | |  |
| *Please initial each box* | | | | | |
| 1. I confirm that I have read and understand the patient information sheet date: …………… (Version: ............) describing the above study and have had the opportunity to consider the information, ask questions and have had these answered satisfactorily. | | | | |  |
| 2. I understand that my participation in this study is voluntary and that I am free to withdraw at any time without giving a reason, without my medical care or legal rights being affected. | | | | |  |
| 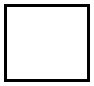3. I understand that sections of my medical notes and data collected during the study may be looked at by responsible individuals involved in this research or from regulatory authorities where it is relevant to my taking part in research. I give permission for these individuals to have access to my records | | | | |  |
| 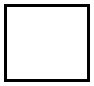4. I agree to allow my General Practitioner and any other relevant medical practitioner to be informed of my involvement in the study. | | | | |  |
| 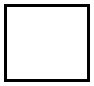5. I agree for a copy of this completed consent form to be sent to the Liverpool Cancer Trial Unit (where it will be kept in a secure location), to allow confirmation that my consent for the trial has been given | | | | |  |
| 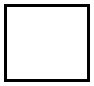6. I agree to take part in the above study | | | | |  |
| 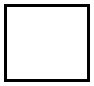7. I understand that information held by the NHS and records maintained by the NHS Information Centre may be used to keep in touch with me and follow-up my health status. | | | | |  |
| 8. I give permission for swabs to be taken from my mouth and neck wound and transferred to the University of Liverpool for research into the prevention and treatment of infections. I understand that nobody outside of the study will have access to them and that they will be destroyed at the end of the study. | | | | | *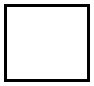* |
|  | | | | |  |
| Name of patient |  | Date |  | Signature | |
|  |  |  |  |  | |
| Name of person taking consent (if different from researcher) |  | Date |  | Signature | |
|  |  |  |  |  | |
| Researcher |  | Date |  | Signature | |
